# Effect of Sperm Concentration and Storage Temperature on Goat Spermatozoa during Liquid Storage

**DOI:** 10.3390/biology9090300

**Published:** 2020-09-19

**Authors:** Sara Sadeghi, Raquel Del Gallego, Balma García-Colomer, Ernesto A. Gómez, Jesús L. Yániz, Jaime Gosálvez, Carmen López-Fernández, Miguel A. Silvestre

**Affiliations:** 1 Departamento de Biología Celular, Biología Funcional y Antropología Física, Universitat de València, 46100 Valencia, Spain; sara.sadeghi1@gmail.com (S.S.); radelgabo@gmail.com (R.D.G.); balgarco@alumni.uv.es (B.G.-C.); 2 Centro de Investigación y Tecnología Animal, Instituto Valenciano de Investigaciones Agrarias, Pol. La Esperanza 100, 12400 Castellón, Spain; gomez_ern@gva.es; 3 BIOFITER Research Group, Institute of Environmental Sciences (IUCA), University of Zaragoza, 22071 Huesca, Spain; jyaniz@unizar.es; 4 Department of Biology, Universidad Autónoma de Madrid, 28049 Madrid, Spain; jaime.gosalvez@uam.es (J.G.); mariadelcarmen.lopez@uam.es (C.L.-F.)

**Keywords:** goat sperm, liquid storage, motility, DNA fragmentation, mitochondrial activity

## Abstract

**Simple Summary:**

One of the problems with the refrigerated storage of spermatozoa in goats is the short shelf life. The study of the effect of the different storage conditions on the different sperm parameters could help to increase sperm survival. In this work, we studied the effect of temperature and sperm concentration during refrigerated storage on sperm motility, mitochondrial membrane potential and DNA fragmentation. We observed that refrigerated storage of semen doses reduced the majority of sperm quality parameters, however, sperm DNA fragmentation was not affected. Storage at 5 °C preserved higher sperm motility than at 17 °C. Moreover, the reduction of sperm concentration below 500 × 10^6^ sperm/mL did not seem to improve the quality of spermatozoa.

**Abstract:**

The use of cooled semen is relatively common in goats. There are a number of advantages of cooled semen doses, including easier handling of artificial insemination (AI) doses, transport, more AI doses per ejaculate, and higher fertility rates in comparison with frozen AI doses. However, cooled semen has a short shelf life. The objective of this study was to examine the effect of temperature and sperm concentration on the in vitro sperm quality during liquid storage for 48 h, including sperm motility and kinetics, response to oxidation, mitochondrial membrane potential (MMP) and DNA fragmentation in goats. Three experiments were performed. In the first, the effects of liquid preservation of semen at different temperatures (5 °C or 17 °C), durations (0, 24 and 48 h) and sperm concentrations (250 × 10^6^ sperm/mL (1:2 dilution rate), 166.7 × 10^6^ sperm/mL (1:3 dilution rate) or 50 × 10^6^ sperm/mL (1:10 dilution rate)) on sperm motility and kinetics were studied. In the second experiment, the effect of temperature, sperm washing and concentration on sperm motility and DNA fragmentation was studied. Finally, the effect of sperm concentration and duration of storage at 5 °C on sperm motility, response to oxidative stress and MMP was examined. We found that refrigerated liquid storage of goat sperm impaired sperm quality, such as motility, MMP and response to oxidation, as storage time increased; however, sperm DNA fragmentation index was not significantly affected. Liquid storage at 5 °C preserved higher total motility than at 17 °C. Moreover, we observed that the reduction of sperm concentration below 500 × 10^6^ sperm/mL did not seem to improve the quality of spermatozoa conserved in milk-based extender in the conditions tested.

## 1. Introduction

Artificial insemination (AI) using semen from elite males is key for genetic improvement programmes, both for the evaluation of young males, mainly through their daughters’ performances (progeny test) and for the dissemination of top-ranked tested males. The storage of frozen semen is important both for the establishment of germplasm banks and for the long-term maintenance of doses of valuable breeding males. The use of cooled semen is relatively common in goats, and females are usually inseminated out of the breeding season with cooled semen within the first 24 h of storage [1]. The advantages and disadvantages of AI with cooled semen samples in dairy cattle have been recently reviewed [2]; most of them are similar in the goat. The advantages include easier handling of cooled AI doses, transport and higher fertility rates compared to frozen AI doses [2,3]. Moreover, cooled doses require less spermatozoa per dose, which means more AI doses per ejaculate [4]. Another reason which is becoming more relevant for goats is that the cost of a refrigerated cooled AI dose is lower than the cost of a frozen dose [2]. In goats, it is not always possible to pass through the cervix and to carry out an intrauterine AI. In a previous study, we observed that it was possible to reach and place the semen in the uterus body in only 18% of inseminations, and in 75% of AIs, spermatozoa were placed in the cervical canal [5]. This fact is important since the fertility of cooled doses after AI does not depend so much on the place of semen deposition as it does for the frozen doses [5,6]. Moreover, frozen–thawed goat semen presented lower longevity than chilled semen [7].

The principal disadvantage of chilled semen is its short shelf life [2], which makes it unfeasible for long storage periods or transportation over long distances. The lowering of the storage temperature below body temperature is a common strategy to reduce cellular metabolism [8] and aims to increase storage time. However, hypothermia could also provoke other detrimental effects in animal cells, mainly on sodium homeostasis [8] as, for example, at 5 °C, the Na^+^/K^+^ pump reduces its activity by increasing intracellular Na^+^ levels [9,10].

Conversely, the reduction of sperm concentration in chilled insemination doses, as well as its combination with different storage temperatures, has recently been studied in cattle [4,9,11]. The reduction of bull sperm concentration seems to be beneficial for the fertilisation potential of sperm in vitro, reducing its oxidative stress [4]. In addition, detrimental effects of seminal plasma on the refrigerated storage of horse semen samples can be diminished with higher dilution rates [12,13,14]. In contrast, in rabbits, higher sperm concentrations reported the highest total motility and the lowest rate of viable sperm with low intracellular H_2_O_2_ after 48 h of cooled storage [15]. However, for goats, few studies that focused on the effect of sperm concentration and the temperatures of cooled semen storage were found in the literature. Sperm concentration in goat AI with refrigerated semen is not well established, and a wide range of sperm concentrations, from 800 to 100 × 10^6^ sperm/mL for cooled semen, have been used [16,17,18,19]. In previous works, we have tried to improve the in vitro fertility of refrigerated goat semen, either preserving it in a solid phase or adding antioxidants, but without increasing fertility rates [18]. Over the past few years, it does not appear that significant progress has been achieved for liquid semen storage in goats. Recently, it has been observed that the rates of oxidised viable sperm in cattle and horses after H_2_O_2_ treatment, as sperm respond to oxidation and mitochondrial membrane potential (MMP), among others, were positively correlated to fertility [20,21]. However, little information about these parameters can be found in goats.

Therefore, the purpose of the current study was to examine the effect of temperature and sperm concentration during liquid cooled storage of goat spermatozoa for 48 h on the in vitro spermatozoa quality, including sperm motility and kinetics, response to oxidation, MMP and DNA fragmentation.

## 2. Materials and Methods

### 2.1. Semen Collection

Adult Murciano-Granadina male goats (approximately from 2 to 9 years old) were reared and trained at the Centro de Investigación y Tecnología Animal, Instituto Valenciano de Investigaciones Agrarias (CITA-IVIA, 39°51′55′′ N, 0°30′07′′ W; Segorbe, Spain). CITA-IVIA was approved in accordance with Directive 92/65/EEC for the collection, processing, preservation and storage of semen (registration number: ES17RS01OC). Semen was collected by means of a pre-warmed artificial vagina as in previous studies [18,22] from the end of November to May. Ejaculates were diluted in skimmed milk supplemented with 2 mg/mL glucose (SMG) at a final concentration of approximately 500 × 10^6^ sperm/mL determined by a photometer (Accucell, IMV Technologies, l’Aigle, France) [3]. Semen samples were transported to the laboratory at room temperature within 45 min of collection. Depending on the study, each male ejaculate was kept individually or a pool of three different ejaculates was established.

### 2.2. Sperm Preparation and Experimental Design

To achieve the proposed objective, three experiments were performed.

**Experiment** **1.** Effect of liquid preservation of semen at different temperatures, durations and sperm concentrations on sperm motility and kinetics.

Semen preservation was studied under different conditions. Each individual male ejaculate was diluted in the SMG medium at three sperm concentrations (250 × 10^6^ sperm/mL (1:2 dilution rate), 166.7 × 10^6^ sperm/mL (1:3 dilution rate) or 50 × 10^6^ sperm/mL (1:10 dilution rate)) and stored at different temperatures (5 °C or 17 °C) for 48 h. Sperm were evaluated at three different incubation times (0, 24 and 48 h).

**Experiment** **2.** Effect of temperature, sperm washing and concentration on sperm motility and DNA fragmentation.

A pool from three diluted ejaculates from different males was established. Half of the pool was washed, by centrifugation at 1000× *g* for 10 min, to examine whether the removal of seminal plasma improved the preservation of sperm. As previously described, sperm samples, washed or not, were further diluted in SMG medium at two sperm concentrations (250 × 10^6^ sperm/mL (1:2) or 50 × 10^6^ sperm/mL (1:10)) determined by a photometer. Sperm samples of all sperm concentrations were conserved at two different temperatures (5 °C or 17 °C) for 48 h. Sperm were evaluated at two different times (0 and 48 h). After the motility assessment at 48 h, all the samples were centrifuged, re-suspended in phosphate-buffered saline (PBS) and frozen at −80 °C until all the three replicates were prepared for a DNA fragmentation procedure.

**Experiment** **3.** Effect of sperm concentration and duration of storage at 5 °C on sperm motility, oxidative response and mitochondrial membrane potential.

Sperm samples from a pool were diluted in SMG to three different sperm concentrations (500 × 10^6^ sperm/mL (Control), 250 × 10^6^ sperm/mL (1:2) or 50 × 10^6^ sperm/mL (1:10)) and stored at 5 °C for 48 h. Motility, MMP and oxidation response of spermatozoa were evaluated at 0 and 48 h.

### 2.3. Evaluation of Sperm Samples

#### 2.3.1. Sperm Motility and Kinetics

As described previously [23,24], sperm doses were diluted to approximately 30 × 10^6^ sperm/mL with Tris-BSA medium for their correct evaluation. Following the manufacturer’s recommendation, a volume of 8 µL was then loaded into the droplet-loaded counting chamber Spermtrack^®^ (Proiser R + D S.L., Paterna, Spain), previously warmed to 37 °C. A trinocular UOP microscope with a negative phase contrast objective (×10) and a warmed plate (37 °C) was used. Sperm analysis was performed with the use of the CASA-mot system (ISAS^®^ v1.2; PROISER S.L., Paterna, Spain) using a Basler digital camera (A780-54fm) at a frame rate of 25 images per second. Goat sperm variables were predetermined in the ISAS configuration. Assessment of sperm parameters determined by CASA-mot software included sperm motility (TM, %), progressive motility (PM, %) and kinetic variables: curvilinear velocity (VCL, μm/s), straight-line velocity (VSL, μm/s), average path velocity (VAP, μm/s), straightness (STR = VSL/VAP; %), amplitude of lateral head displacement (ALH, μm) and movement linearity (LIN = VSL/VCL, %). Spermatozoa were classified as progressive if VCL > 45 μm/s and STR > 80%.

#### 2.3.2. DNA Fragmentation Test

The level of sperm DNA fragmentation was assessed using a commercial variant of the sperm chromatin dispersion test (SCD, Halomax^®^; ChromaCell SL, Madrid, Spain [25,26]). After 48 h of storage, all samples were centrifuged at 1000× *g* for 10 min to wash away the skimmed milk, suspended with PBS and stored at −80 °C until SCD analysis, which was performed following the manufacturer’s instructions. All slides were visualised and counted with fluorescence microscopy and at least 300 spermatozoa were evaluated per sample. The sperm DNA fragmentation index (sDFI) was expressed as a percentage.

#### 2.3.3. Mitochondrial Membrane Potential

Mitochondrial membrane potential of spermatozoa was measured by Easykit 2 (Ref. 024864; IMV Technologies, l’Aigle, France [20]). Results were expressed as two groups: sperm with low (depolarised) or high mitochondrial membrane potential (hMMP; polarised).

#### 2.3.4. Response to Oxidative Stress

The reactive oxygen species level was measured by Easykit 3 (Ref. 025157; IMV Technologies, l’Aigle, France [20]). In this test, H_2_O_2_ treatment (Ht) was performed [20]. Results were expressed as four groups: oxidised or not, and sperm plasma membrane—intact (viable) or damaged. We evaluated oxidised sperm (ROS+) after Ht from the total and viable populations.

#### 2.3.5. Flow Cytometry

The mitochondrial status and the oxidation response test were evaluated using a BD LSRFortessa flow cytometer. These analyses were performed by the cell culture and flow cytometry section of the Central Service for Experimental Research (SCSIE) of the University of Valencia. The flow cytometer contains four excitation lasers: UV 355 nm, blue 488 nm, yellow-green 561 nm, and red 640 nm. A minimum of 3000 cells/replicate and group were assessed.

### 2.4. Statistical Analysis

At least three replicates were performed in each experiment. Prior to the statistical analyses, depending on the dependent variable, non-percentage (VCL, VSL, VAP and LIN) or percentage (TM, PM, sDFI, hMMP, ROS+), a log or arcsine of the square root transformation was performed, respectively. Generalised linear model analysis was used (IBM SPSS Statistics, v26). The main effects and all possible two-way interactions were included in the models depending on the experiment. The results of the main effects are shown as mean ± standard error. For Experiment 1, we included the effects of male, temperature, dilution, time and double interactions in the model for all variables. For Experiment 2, we included the effects of centrifugation, temperature, dilution, time and double interactions in the model for all variables, except for sDFI. For sDFI at 48 h, we included the effects of centrifugation, temperature, dilution and double interactions in the model. In order to compare sDFI at 0 and 48 h, we only included the effect of time in the model. For Experiment 3, we included the effects of dilution, time and double interaction in the model. A *p*-value of *p* < 0.05 was considered to be statistically significant.

## 3. Results

### 3.1. Effect of Liquid Preservation of Semen at Different Temperatures, Durations and Sperm Concentrations on Sperm Motility and Kinetics

**Figure 1:**
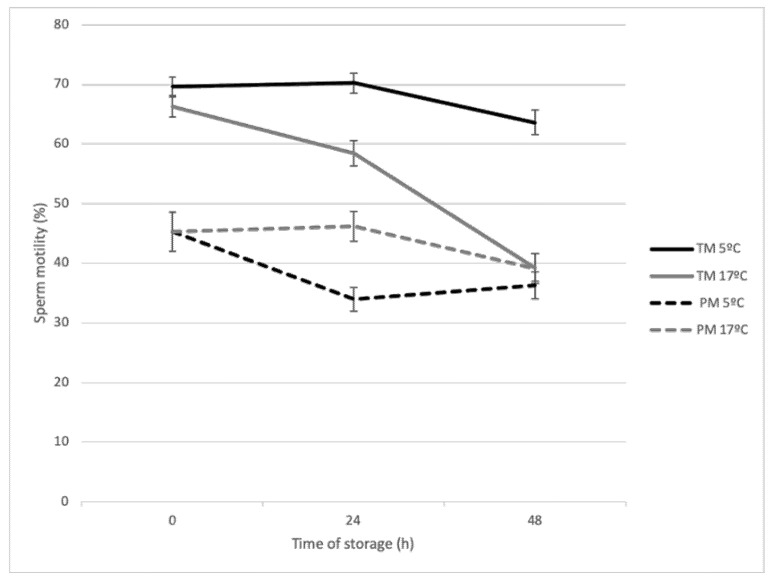
Effect of different temperatures (5 °C or 17 °C) and duration of liquid storage on total (TM) and progressive (PM) sperm motility of goat spermatozoa.

Significance levels and results of the main effects of the model from the first experiment are presented in Table 1 and Table 2 and Figure 1. Sperm total (TM) or progressive motility (PM) and velocities (VCL, VSL and VAP) significantly diminished as storage time increased. Storage time did not affect LIN, STR and ALH sperm parameters (Table 1). The decrease in TM was mainly important when semen was stored at 17 °C (Figure 1; *p* < 0.05). In relation to temperature of storage, sperm stored at 5 °C revealed higher TM, VCL and VAP than sperm stored at 17 °C (67.8 vs. 54.6% for 5 °C and 17 °C, respectively, Table 2, *p* < 0.05) without changes in PM. However, motile sperm preserved at 17 °C had a higher STR rate and higher LIN than sperm stored at 5 °C (*p* < 0.05; Table 1 and Table 2). After 24 h of storage, the sperm samples preserved at 17 °C had a higher PM than at 5 °C, although at 48 h these were similar for both temperatures (*p* < 0.05; Figure 1). Regarding dilution rate, spermatozoa stored at the 1:3 dilution rate presented significantly lower TM than at the 1:10 dilution rate (*p* < 0.05; Table 2). No statistical differences were found in the rest of kinetic parameters of spermatozoa except for ALH (Table 1).

### 3.2. Effect of Temperature, Time, Sperm Washing and Concentration on Sperm Motility and Kinetics and DNA Fragmentation

Significance levels and results from Experiment 2 are presented in Table 3 and Table 4. As in Experiment 1, sperm TM, PM and velocities significantly decreased as cooled storage time increased until 48 h. In this experiment, storage time also reduced LIN and STR sperm parameters. No significant statistical differences were found in sDFI between the sperm samples at 0 h and after storage for 48 h. 

In the case of sperm dilution, no statistical differences for TM and PM were found, however, sperm velocities and ALH were higher in samples stored with higher sperm concentrations (*p* < 0.05; Table 4). Moreover, sDFI was higher when the sperm dilution rate was lower (4.3 vs. 2.7 for sDFI for 1:2 and 1:10 dilution rate; *p* < 0.05; Table 4).

**Figure 2:**
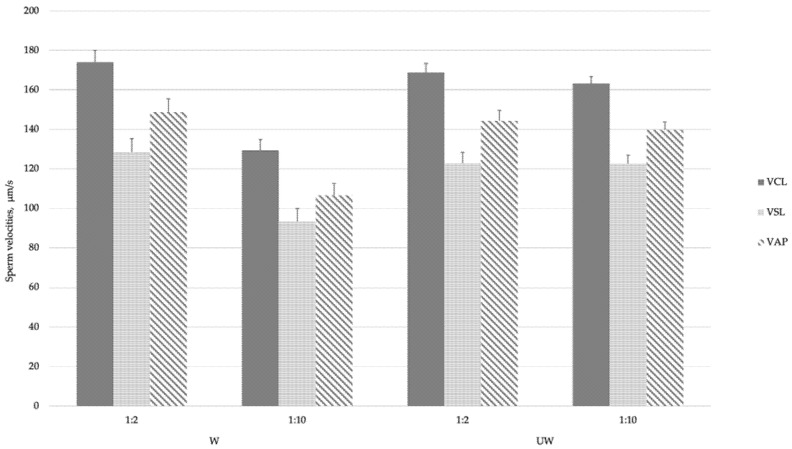
Effect of dilution and washing (washed: W vs. not washed: UW) on velocities (VLC, VSL and VAP) of goat spermatozoa after liquid storage.

Regarding storage temperatures, as in Experiment 1, spermatozoa stored at 5 °C revealed higher TM and ALH and lower LIN than spermatozoa stored at 17 °C (*p* < 0.05; Table 4). Nevertheless, similar results for PM and velocities were observed at both temperatures. No statistical differences were detected in sDFI between samples stored for 48 h for both temperatures, ranging from 3.0 to 4.0%. Nor were significant differences found between the samples at 0 h and after incubation for 48 h. In relation to the washing of seminal plasma, washed samples significantly declined in TM and all velocities without modifying PM, STR, LIN or ALH. The detrimental effect of washing on sperm velocities (VCL, VSL and VAP) was especially noticeable at the lowest dilution (Table 3 and Figure 2). Moreover, sDFI was not significantly affected by the washing process (Table 4).

### 3.3. Effect of Duration of Storage and Sperm Concentration on Motility and Kinetics, Mitochondrial Polarisation and Oxidative Response after Storage at 5 °C

The results of the main effects of Experiment 3 are presented in Table 5. No double interaction was statistically significant. As in the two previous experiments, sperm TM and PM significantly declined as storage time increased until 48 h at 5 °C. Similarly, there was a significant drop in the high MMP rate of spermatozoa as duration of storage increased, mainly for the first 24 h (57.3, 27.0 and 15.4% high MMP for 0, 24 and 48 h, respectively; *p* < 0.05; Table 5). Similar results were observed for sperm response to oxidative stress; ROS+ from viable sperm after Ht decreased as storage time increased (33.4, 19.1 and 7.0% ROS+ from viable sperm after Ht for 0, 24 and 48 h, respectively; *p* < 0.05; Table 5).

With regard to sperm dilution, the sperm sample from the Control Group revealed a higher TM than other sperm samples that were more diluted (45.4 vs. 34.5 and 32.6% for Control, 1:2 and 1:10 Groups, respectively; *p* < 0.05; Table 5). However, no significant difference between dilution groups was observed in PM. In relation to mitochondrial activity, no significant differences were found in MMP between the different dilution groups, ranging from 30.3 to 41.5% hMMP. Regarding response to oxidative stress, ROS+ after Ht significantly dropped as sperm dilution was greater than the Control Group, mainly at 1:10 (39.0 vs. 17.0 and 7.0% ROS+ after Ht from viable cells for Control, 1:2 and 1:10 Dilution Groups, respectively; *p* < 0.05; Table 5). 

## 4. Discussion

In this study, our objective was to examine the effect of sperm concentration and temperature during liquid storage of goat semen on motility, mitochondrial activity and DNA fragmentation of spermatozoa. As noted above, the main objection to cooled storage is the short shelf life. In goats, as found in previous work, we observed that sperm motility declined as time increased [18]. Furthermore, we observed that other quality parameters of goat spermatozoa stored at 5 °C, such as velocities and hMMP, also decreased as storage time increased. Recently, other authors also observed a gradual decrease in velocities and hMMP as the length of sperm storage time at 5 °C increased [27,28,29,30,31]. Moreover, Liu et al. [28] also reported the detrimental effect of goat spermatozoa storage at 4 °C in other quality parameters, showing an increase in apoptosis and defects of mitochondria as time increased. On the other hand, response to oxidative stress has been used as a sperm quality parameter since it was positively correlated with fertility [20,21]. We observed that response to oxidative stress also diminished as liquid storage time increased, presuming that sperm stored for 48 h would have a lower fertilisation potential. However, no more information in the literature was found. One strategy to extend the shelf life of spermatozoa is a reversible reduction of metabolic activity. Hypothermia is able to reduce metabolic activity of cells by decelerating enzymatic reactions [10,32,33]. In this study, we tested two temperatures commonly used in liquid preservation of semen. We observed that goat sperm stored at 5 °C retained higher TM than at 17 °C, but had lower LIN. In this way, other authors also observed that spermatozoa stored at 4–5 °C showed greater total motility rates than at higher temperatures (17–20 °C) in goats [34,35], and also in sheep [36,37]. Conversely, Qiu et al. [38] observed greater motility in goat spermatozoa stored at 15 °C in comparison to 5 °C or 25 °C. However, they only used PBS without any supplement as a semen extender, while in the present work, we used a skimmed milk extender with glucose. It is known that the harmful effects of cold shock during storage of sperm could be mitigated using skimmed milk as an extender or supplementation [39,40]. In fact, in our laboratory, we also observed that spermatozoa conserved in a less complex medium such as Tris-citric-glucose-BSA revealed a higher sperm motility, both total and progressive, at 17 °C in comparison with 5 °C [41]. 

In the present study, no effect of cooled storage time on sDFI was observed, regardless of storage temperature, which is in agreement with Murphy et al.’s [9] findings in cattle. No information about the effect of cooled liquid preservation in goat sperm DNA fragmentation was found. Linfor and Meyers [42] indicated that equine sperm DNA damage was not significantly increased until after 48 h of storage. In other studies, sDFI increased as cooled storage time increased beyond 48 h in equine spermatozoa [43,44]. In porcine spermatozoa, sDFI did not change during the first five days at 15 °C [45]. In our experiment, sperm samples were only cool stored for 48 h, and this time period at both temperatures might not be enough to appreciably damage DNA in goat sperm.

Sperm washing is a normal procedure that is used to split seminal plasma and spermatozoa to avoid its detrimental interaction with egg yolk or milk-based extender in goat sperm cryopreservation [46]. An increase in *g*-force and/or duration of the centrifugation process minimises sperm loss [47]. However; the mechanical actions of centrifugation and spermatozoa compaction at the bottom could reduce sperm quality [48]. We observed that TM and velocities of spermatozoa declined after sperm washing, in agreement with Marzano et al. [49] in equine sperm. In a previous study, we observed a beneficial effect of sperm washing on motility in goats [18] without a significant deleterious effect of the centrifugation process, in agreement with others [47]. It is possible that the holding time before sperm washing was sufficient to reduce goat sperm motility and viability during storage at 5 °C in the present experiment [50]. In bovines, we also observed a detrimental effect of centrifugation on sperm motility [51]. In the present study, no effect of the sperm centrifugation process on sDFI was detected, in agreement with other studies in humans, equines and goats [47,52,53].

With regard to sperm concentration, the beneficial effect of storing goat sperm doses at low sperm concentration on sperm motility and kinetics was not evident, as observed by other research groups [4,9,11]. Different results of sperm motility were obtained in the different experiments of the present study. In the last experiment, we observed that Control Group showed a significantly higher TM than Dilution Groups and a continuous drop in the hMMP as the sperm concentration rate was lowered. In agreement with our results, other authors [9,31,54] observed that higher sperm concentration during liquid storage maintained higher motility and hMMP rates in ovines. In contrast, Murphy et al. [4] found that bovine spermatozoa stored at lower concentrations showed greater viability and reduced glucose consumption. However, no effect of sperm concentration on sperm PM and hMMP was found [4,11]. This disagreement could be attributed to the use of different species and methodology, and that the fact that they did not use any CASA system for sperm motility assessment. Moreover, other factors could affect the final results since even the use of different breeds or extenders had an effect on the hMMP of spermatozoa [27,55]. With regard to the effect of sperm concentration on DNA fragmentation, we observed that sDFI was higher when sperm concentration increased. It was observed that DNA longevity depends largely on sperm concentration during incubation [56]. After incubation at 37 °C, the DNA fragmentation rate was greater in samples with high sperm concentration, but it had a significant individual effect [56,57]. One explanation could be that an increase in oxidative stress (e.g., H_2_O_2_) occurs when spermatozoa are stored at high concentrations [4,15], and it is known that an increase in oxidative stress induces nuclear and mitochondrial DNA damage [58]. However, other authors found lower sDFI in more concentrated samples [15,31,54] or no effect of sperm concentration [9]. Finally, response to oxidative stress was reduced as sperm concentration decreased, which could mean that among the sperm concentrations tested, that of Control Group may be the most appropriate for liquid storage of goat sperm.

## 5. Conclusions

In conclusion, we found that refrigerated liquid storage of goat sperm impaired sperm quality, such as motility, MMP and response to oxidation, as conservation time increased; however, sDFI was not affected. Liquid storage at 5 °C preserved higher total motility than at 17 °C. Moreover, we observed that reduction of sperm concentration below 500 × 10^6^ sperm/mL did not seem to improve the quality of spermatozoa conserved in a milk-based extender in the tested conditions.

## Figures and Tables

**Table 1:** Significance levels of factors for variables studied in Experiment 1.

Factors ^1^	Variables ^2^							
	TM	PM	VCL	VSL	VAP	LIN	STR	ALH
Time	**	**	**	**	**	NS	NS	NS
Temperature	**	NS	**	NS	**	*	**	NS
Dilution	**	NS	NS	NS	NS	NS	NS	*
Ti*Tp	**	*	NS	**	*	**	**	**
Ti*Di	NS	NS	NS	NS	NS	NS	NS	NS
Tp*Di	NS	NS	*	**	*	NS	NS	NS

^1^ Di: dilution rate; Ti: time; Tp: temperature. ^2^ TM: total motility, %; PM: progressive motility, %; VCL: curvilinear velocity, μm/s; VSL: straight-line velocity, μm/s; VAP: average path velocity, μm/s; LIN: linearity of the curvilinear trajectory, %; STR: straightness, %; ALH: amplitude of lateral head displacement, μm. NS: no significant differences; * *p* < 0.05; ** *p* < 0.01.

**Table 2:** Effect of semen liquid preservation at different temperatures, durations and sperm concentrations on sperm motility and kinetics.

Factors ^1^	Variables ^2^							
	TM	PM	VCL	VSL	VAP	LIN	STR	ALH
Temperature								
5 °C	67.8 ± 1.0 ^a^	38.5 ± 1.5	138.1 ± 1.6 ^a^	74.6 ±1.5	118.2 ± 1.3 ^a^	58.6 ± 1.0 ^b^	65.6 ± 1.0 ^b^	3.7 ± 0.1
17 °C	54.6 ± 1.4 ^b^	43.5 ± 1.6	129.7 ± 1.8 ^b^	77.0 ± 1.5	110.5 ± 1.6 ^b^	62.2 ± 0.9 ^a^	71.2 ± 0.9 ^a^	3.7 ± 0.1
Dilution								
1:2	61.2 ± 1.8 ^ab^	38.7 ± 2.0	134.1 ± 2.3	74.8 ± 1.8	115.8 ± 2.0	59.4 ± 1.2	66.8 ± 1.2	3.6 ± 0.1 ^b^
1:3	57.6 ± 1.6 ^b^	41.8 ± 2.0	131.9 ± 2.2	74.3 ± 1.9	112.2 ± 1.9	60.2 ± 1.3	68.3 ± 1.2	3.7 ± 0.1 ^ab^
1:10	64.9 ± 1.4 ^a^	42.5 ± 1.9	135.9 ± 2.0	78.4 ± 1.9	115.0 ± 1.7	61.6 ± 1.2	70.2 ± 1.1	3.9 ± 0.1 ^a^
Time (h)								
0	67.9 ± 1.2 ^a^	45.3 ± 2.3 ^a^	148.4 ± 1.6 ^a^	85.6 ± 2.3 ^a^	127.0 ± 1.5 ^a^	61.4 ± 1.5	68.1 ± 1.4	3.9 ± 0.1
24	64.3 ± 1.4 ^a^	40.0 ± 1.7 ^ab^	132.0 ± 1.5 ^b^	73.6 ± 1.5 ^b^	113.6 ± 1.2 ^b^	59.4 ± 1.2	66.8 ± 1.1	3.7 ± 0.1
48	51.4 ± 1.8 ^b^	37.7 ± 1.7 ^b^	121.6 ± 2.5 ^c^	68.4 ± 1.4 ^b^	102.6 ± 2.2 ^c^	60.5 ± 0.9	70.2 ± 1.0	3.6 ± 0.1

^1^ Dilution rate: (250 × 10^6^ sperm/mL (1:2 dilution rate), 167 × 10^6^ sperm/mL (1:3 dilution rate) or 50 × 10^6^ sperm/mL (1:10 dilution rate)). Time: 0 h refers to values obtained within 1 h after semen collection. ^2^ TM: total motility, %; PM: progressive motility, %; VCL: curvilinear velocity, μm/s; VSL: straight-line velocity, μm/s; VAP: average path velocity, μm/s; LIN: linearity of the curvilinear trajectory, %; STR: straightness, %; ALH: amplitude of lateral head displacement, μm. Different superscripts ^(a–c)^ within the same column indicate significant differences (*p* < 0.05).

**Table 3:** Significance levels of factors for variables studied in Experiment 2.

Factors ^1^	Variables ^2^								
	sDFI ^3^	TM	PM	VCL	VSL	VAP	LIN	STR	ALH
Time	NS	**	**	**	**	**	**	**	**
Temperature	NS	**	NS	NS	NS	NS	**	NS	**
Washing	NS	**	NS	**	**	**	NS	NS	NS
Dilution	*	NS	NS	**	**	**	NS	NS	**
Ti*Tp	NS	**	NS	**	NS	**	**	NS	**
Ti*Wh	-	NS	**	**	**	**	**	**	NS
Ti*Di	-	NS	NS	NS	NS	NS	*	*	NS
Tp*Wh	NS	NS	*	*	*	*	**	**	NS
Tp*Di	*	NS	*	NS	NS	NS	NS	*	NS
Wh*Di	NS	NS	NS	**	**	**	NS	NS	**

^1^ Di: dilution rate; Ti: time; Tp: temperature; Wh: washing. ^2^ sDFI: sperm DNA fragmentation index, %, TM: total motility, %; PM: progressive motility, %; VCL: curvilinear velocity, μm/s; VSL: straight-line velocity, μm/s; VAP: average path velocity, μm/s; LIN: linearity of the curvilinear trajectory, %; STR: straightness, %; ALH: amplitude of lateral head displacement, μm. ^3^ For sDFI at 48 h, we included the effects of Wh, Tp, Di and double interactions in the model. In order to compare sDFI at 0 and 48 h, we only included the effect of Ti in the model. NS: no significant differences; * *p* < 0.05; ** *p* < 0.01.

**Table 4:** Effect of temperature, time, sperm washing and concentration on sperm motility and kinetics and DNA fragmentation after liquid storage.

Factors ^1^	Variables ^2^								
	sDFI	TM	PM	VCL	VSL	VAP	LIN	STR	ALH
Centrifugation									
W	3.6 ± 0.6	58.6 ± 2.9 ^b^	59.8 ± 2.6	151.4 ± 4.7 ^b^	110.6 ± 5.1 ^b^	127.4 ± 5.0 ^b^	68.0 ± 1.9	80.6 ± 1.3	3.3 ± 0.1
NW	3.5 ± 0.7	66.6 ± 2.3 ^a^	63.2 ± 1.4	166.2 ± 2.9 ^a^	122.8 ± 3.4 ^a^	142.1 ± 3.3 ^a^	69.8 ± 1.3	81.6 ± 0.8	3.4 ± 0.1
Temperature									
5 °C	4.0 ± 0.7	71.5 ± 1.2 ^a^	58.7 ± 1.7	155.5 ± 2.6	106.5 ± 3.4	125.4 ± 3.1	65.2 ± 1.6 ^b^	80.0 ± 1.0	3.7 ± 0.1 ^a^
17 °C	3.0 ± 0.4	53.7 ± 3.3 ^b^	64.7 ± 2.4	162.5 ± 5.1	128.0 ± 5.1	145.1 ± 5.3	72.9 ± 1.5 ^a^	82.3 ± 1.1	3.0 ± 0.1 ^b^
Dilution									
1:2	4.3 ± 0.7 ^a^	63.8 ± 2.6	61.7 ± 1.9	171.4 ± 3.7 ^a^	125.5 ± 4.3 ^a^	146.4 ± 4.2 ^a^	69.0 ± 1.5	80.9 ± 0.9	3.5 ± 0.1 ^a^
1:10	2.7 ± 0.5 ^b^	61.5 ± 2.7	61.4 ± 2.2	145.9 ± 3.7 ^b^	107.6 ± 4.3 ^b^	122.7 ± 4.1 ^b^	68.8 ± 1.8	81.2 ± 1.2	3.2 ± 0.1 ^b^
Time (h)									
0	5.5 ± 1.3	76.3 ± 1.0 ^a^	72.6 ± 0.9 ^a^	172.6 ± 3.1 ^a^	143.8 ± 2.7 ^a^	157.6 ± 3.0 ^a^	79.8 ± 0.7 ^a^	87.5 ± 0.5 ^a^	2.8 ± 0.0 ^b^
48	3.5 ± 0.4	48.9 ± 3.0 ^b^	49.3 ± 2.3 ^b^	143.6 ± 4.2 ^b^	86.8 ± 3.8 ^b^	109.6 ± 4.1 ^b^	56.9 ± 1.5 ^b^	73.9 ± 1.0 ^b^	4.0 ± 0.1 ^a^

^1^ Dilution rate: (250 × 10^6^ sperm/mL (1:2 dilution rate) or 50 × 10^6^ sperm/mL (1:10 dilution rate)). Time: 0 h refers to values obtained within 1 h after semen collection. Centrifugation: samples that had their seminal plasma washed (W) and not washed (NW). ^2^ sDFI: sperm DNA fragmentation index, %; TM: total motility, %; PM: progressive motility, %; VCL: curvilinear velocity, μm/s; VSL: straight-line velocity, μm/s; VAP: average path velocity, μm/s; LIN: linearity of the curvilinear trajectory, %; STR: straightness, %; ALH: amplitude of lateral head displacement, μm. Different superscripts ^(a,b)^ within the same column indicate significant differences (*p* < 0.05).

**Table 5:** Effect of duration of storage and sperm concentration on motility and kinetics, mitochondria polarisation and oxidative response after storage at 5 °C.

Factors ^1^	Variables ^2^				
	TM	PM	hMMP	ROS + from TS	ROS + from VS
Dilution					
Control	45.4 ± 4.6 ^a^	85.1 ± 3.2	41.5 ± 8.6	38.4 ± 4.8 ^a^	39.0 ± 4.7 ^a^
1:2	34.5 ± 4.8 ^b^	82.5 ± 3.9	32.8 ± 8.4	16.0 ± 5.2 ^b^	17.0 ± 5.1 ^b^
1:10	32.6 ± 4.4 ^b^	76.2 ± 4.7	30.3 ± 8.7	6.1 ± 3.4 ^c^	7.0 ± 3.4 ^c^
Time (h)					
0	71.1 ± 1.7 ^a^	93.0 ± 1.2 ^a^	57.3 ± 3.9 ^a^	32.4 ± 6.0 ^a^	33.4 ± 5.6 ^a^
24	34.3 ± 3.2 ^b^	78.0 ± 3.5 ^b^	27.0 ± 7.3 ^b^	18.1 ± 5.1 ^b^	19.1 ± 5.1 ^b^
48	6.1 ± 1.5 ^c^	65.2 ± 7.2 ^b^	15.4 ± 8.9 ^b^	6.6 ± 4.0 ^c^	7.0 ± 4.1 ^c^

^1^ Dilution rate: (500 × 10^6^ sperm/mL (Control), 250 × 10^6^ sperm/mL (1:2 dilution rate) or 50 × 10^6^ sperm/mL (1:10 dilution rate)). Time: 0 h refers to values obtained within 1 h after semen collection. ^2^ TM: total motility, %; PM: progressive motility, %; hMMP: sperm with high mitochondrial membrane potential, %; ROS+: oxidised sperm after H_2_O_2_ treatment, %; TS: total sperm; VS: viable sperm. Different superscripts ^(a–c)^ within the same column indicate differences in pairwise comparisons *(p* < 0.05).
